# An analysis of complaints about hospital care in the Republic of Ireland

**DOI:** 10.1093/intqhc/mzac037

**Published:** 2022-05-12

**Authors:** Emily O’dowd, SinÉad Lydon, Kathryn Lambe, Akke Vellinga, Chris Rudland, Elaine Ahern, Aoife Hilton, Marie E Ward, Maria Kane, Tom Reader, Alex Gillespie, David Vaughan, Dubhfeasa Slattery, Paul O’connor

**Affiliations:** Discipline of General Practice, School of Medicine, National University of Ireland Galway, Galway H91TK33, Ireland; Irish Centre for Applied Patient Safety and Simulation, School of Medicine, National University of Ireland Galway, Galway, Ireland; Irish Centre for Applied Patient Safety and Simulation, School of Medicine, National University of Ireland Galway, Galway, Ireland; School of Medicine, National University of Ireland Galway, Galway, Ireland; Health Research Board, Dublin D02 H638, Ireland; School of Public Health, Physiotherapy and Sport Science, University College Dublin D04 V1W8, Ireland; National Complaints Governance and Learning Team, Health Service Executive, Catherine Street, Limerick V94 AY27, Ireland; National Complaints Governance and Learning Team, Health Service Executive, Catherine Street, Limerick V94 AY27, Ireland; National Complaints Governance and Learning Team, Health Service Executive, Catherine Street, Limerick V94 AY27, Ireland; Quality and Safety Improvement Directorate, St James’s Hospital, Dublin D08 NHY1, Ireland; Quality and Safety Improvement Directorate, St James’s Hospital, Dublin D08 NHY1, Ireland; Centre for Innovative Human Systems, School of Psychology, Trinity College, The University of Dublin, Dublin D02 PN40, Ireland; London School of Economics, London, UK; London School of Economics, London, UK; Department of Paediatrics, Children’s Health Ireland, Tallaght, Dublin, Ireland; Health Service Executive, Dublin, Ireland; Discipline of General Practice, School of Medicine, National University of Ireland Galway, Galway H91TK33, Ireland; Irish Centre for Applied Patient Safety and Simulation, School of Medicine, National University of Ireland Galway, Galway, Ireland

**Keywords:** quality improvement, hospitals, patient safety, statistical data analysis, patient satisfaction, patient-centred care

## Abstract

**Background:**

Patients and family members make complaints about their hospital care in order to express their dissatisfaction with the care received and prompt quality improvement. Increasingly, it is being understood that these complaints could serve as important data on how to improve care if analysed using a standardized tool. The use of the Healthcare Complaints Analysis Tool (HCAT) for this purpose has emerged internationally for quality and safety improvement. Previous work has identified hot spots (areas in care where harm occurs frequently) and blind spots (areas in care that are difficult for staff members to observe) from complaints analysis. This study aimed to (i) apply the HCAT to a sample of complaints about hospital care in the Republic of Ireland (RoI) to identify hot spots and blind spots in care and (ii) compare the findings of this analysis to a previously published study on hospital complaints in the UK.

**Methods:**

A sample of complaints was taken from 16 hospitals in the RoI in Quarter 4 of 2019 (*n = *641). These complaints were coded using the HCAT to classify complaints by domain, category, severity, stage of care and harm. Chi-squared tests were used to identify hot spots, and logistic regression was used to identify blind spots. The findings of this study were compared to a previously published UK study that used HCAT to identify hot spots and blind spots.

**Results:**

Hot spots were identified in Irish hospital complaints while patients were receiving care on the ward, during initial examination and diagnosis, and while they were undergoing operations or procedures. This aligned with hot spots identified in the UK study. Blind spots were found for systemic problems, where patients experience multiple issues across their care.

**Conclusions:**

Hot spots and blind spots for patient harm can be identified in hospital care using the HCAT analysis. These in turn could be used to inform improvement interventions, and direct stakeholders to areas that require urgent attention. This study also highlights the promise of the HCAT for use across different healthcare systems, with similar results emerging from the RoI and the UK.

## Introduction

Healthcare complaints are formal expressions of dissatisfaction with the care provided to patients [1]. They are typically communicated through a letter by a patient or their family [2]. Patient insights, as derived from complaints, are increasingly recognized as a useful source of data on how to improve care [3]. In order to promote an equitable, patient-centred health service, it is vital that we incorporate the patient perspective and extend the same level of respect to their input as is offered to healthcare providers and managers working within the system [3]. However, research suggests that although complaints about a unit can prompt changes within that unit, typically, the learning from complaints is not shared externally [4]. Accordingly, there is potential for complaints data to be used in the aggregate for quality and safety improvement in healthcare [1].

The Healthcare Complaints Analysis Tool (HCAT) has been developed to provide a systematic, reliable method of accessing and analysing patient complaints about secondary care [5] and is intended to support the analysis of and learning from complaints and facilitate care improvement. The HCAT can be used to identify three distinct domains (clinical, management and relationship) within healthcare complaints, comprising seven categories (safety, quality, environment, institutional processes, listening, communication and respect and patient rights). The HCAT also categorizes the stage of care, harm and severity reported in the complaint [5].

The HCAT has been used to analyse patient complaints in a number of countries globally (all of which are considered developed economically) [5–9]. The tool has been found to remain reliable when applied in different health systems when used on claims for compensation and when translated [9–11]. It has demonstrated the potential to assist stakeholders in identifying areas in hospital care that require quality improvement [5]. Previous applications of the HCAT have identified ‘hot spots’ (i.e. points in care where there is a high occurrence of harm or near misses where harm could have occurred [12]) and ‘blind spots’ (i.e. areas in care that are not easily witnessed by staff members within an organization or that can be witnessed incorrectly by staff members [12]). It has been proposed that identifying these areas using the HCAT could help with the development of improvement interventions [7, 13].

The aim of our study was to apply the HCAT to patient complaints made about healthcare in the Republic of Ireland (RoI) and identify areas in care where quality improvement is required. This analysis also aims to provide a comparison with a previously published UK study that used the HCAT to analyse complaints in order to provide some context to the data from the RoI complaints [12].

## Method

### Design

This is a retrospective analysis of a database of complaints made by patients about hospitals in the RoI.

### Setting

This study was conducted using complaints about hospital care in the public healthcare system in the RoI.

### Sample

Individual hospitals were contacted by the Health Service Executive (HSE), National Complaints Governance and Learning Team, and asked to contribute their complaints from Quarter 4 of 2019 for analysis. The HSE is the health service in the RoI. Redacted complaints were obtained from 16 hospitals. For the purpose of this study, a ‘complaint’ is considered to be a formal expression of dissatisfaction with healthcare received, made by a patient, family member or other representatives to the hospital involved or the HSE. Claims for financial compensation and disciplinary complaints made to external governing bodies such as the Irish Medical Council were not included.

### Ethical approval

This study received ethical approval from the NUI Galway Research Ethics committee (reference: 18 September 2017).

### Procedure

The complaints were collated using Microsoft Excel. Each complaint was read thoroughly by one of four researchers (K.L., E.O.D., M.E.W. or M.K.), and then, the HCAT was used to categorize the complaints. A total of 25% of complaints were double coded by the second author to ensure inter-rater reliability was sufficient. It was decided to code 25% based on both the timeframe available for the project, as well as previous health research, which has determined 10% as the minimum recommended percentage for inter-rater reliability [14, 15]. The HCAT was applied to categorize individual issues that were reported within complaints in terms of the following:

- the domain of the issue(s)- clinical, management or relationship;
- problem categories of issue(s)—institutional processes, quality, respect and patient rights, communication, safety, environment or listening;
- stage of care at which the issue(s) occurred—admission, examination, ward, operation/procedure, discharge or unknown/other (e.g. when the stage was unclear);
- severity of issue(s)—1 (low) to 3 (high); and
- overall harm reported by the patient in the complaint—0 (no harm/harm not mentioned) to 5 (catastrophic harm).

#### Data preparation

Data were exported from Microsoft Excel into R statistical software [16] for analysis. Data were cleaned, and the ‘harm’ variable was transformed from a variable with six levels to a dichotomous variable. This was required for a subsequent part of the analysis as the sample size was too small for the statistical test to reliably run with a variable with six levels. This action has previously been taken in an analysis of general practice complaints [13] and was the approach recommended by an epidemiologist (A.V.). As a result, any complaint that did not report harm (i.e. harm = 0) was coded as ‘No harm’ and complaints that reported harm of any level (from minor to catastrophic) were coded as containing ‘harm’. While only one level of harm is recorded per complaint, the other aspects of the HCAT are measured at the ‘issue’ level, that is, each problem within a complaint is considered separately.

#### Analysis

The analysis was made up of five distinct parts.

1. *Descriptive analysis*. Following coding, analyses of the features of the complaints were conducted by one researcher (E.O.D.). The characteristics of staff and complainants were captured where possible, and the frequencies of each HCAT aspect were also captured.
2. *Inter-rater reliability.* Complaints were initially coded using the HCAT by one researcher (K.L./E.O.D./M.E.W./M.K.). Following this, 25% of complaints were double coded by a second researcher (either K.L. or E.O.D.). The agreement was calculated using Gwet’s AC1 statistic. This is more robust than the traditionally used kappa statistic [17] and was considered appropriate for the purposes of this study as it has been used previously when employing the HCAT and could therefore be used for comparative purposes [5, 17].
3. *Identification of hot spots*. One of the core aspects of this analysis was to conduct an exploration of the hot spots that emerged. Hot spots are points in care where harm to patients is reported more frequently than would be expected [12]. There can also be hot spots for near misses—where a complaint is coded as high severity; however, no harm was reported, indicating a near-miss incident [12]. Hot spots were analysed using chi-squared tests (for trend), testing the relationship between reported harm and stage of care, following the R script written by Reader and Gillespie [12].
4. *Identification of blind spots*. Blind spots are the next aspect of this complaints analysis. Blind spots are points across care that staff members within a healthcare organization cannot easily measure or observe or indeed that cannot be observed at all [12]. However, patients often witness these issues. There are three types of blind spots. Blind spots for entry and exit into care are identified by computing the number of complaints issues that occur at Stages 1 (Admissions) and 5 (Discharge). Blind spots for errors of omission are defined within the HCAT as issues within the categories ‘Quality’, ‘Listening’ and ‘Communication’ [12]. Blind spots for systemic issues (i.e. where patients experience issues in care across multiple points in the care pathway) were analysed using logistic regression, with harm as the dependent variable, and number of issues and number of stages within a complaint as the independent variables in the model. No confounding variables were included in the model. Similar to the hot spot analysis, this used the code written by Reader and Gillespie [12].
5. *Narrative comparison with UK complaint data.* Data were extracted on key findings from the application of the HCAT to a sample of complaints about hospitals in the UK [12].

## Results

### Descriptive statistics

A total of 641 complaints, pertaining to Quarter 4 of 2019, were analysed. This represented 72% of all complaints (total *n *= 896) received by Irish hospitals in this period and allowed for a 2% margin of error with a 95% confidence level. This means that we can be confident to the 95% level that across all of the complaints received by Irish hospitals, our values will fall within 2% of the values in this paper. Within the sample of complaints, there were a total of 1308 unique issues identified. There was a mean of 2.05 issues per complaint (SD = 1.23) and a range of 1–6 issues. A total of 16 hospitals provided data for this study, with an average of 40 complaints provided by each hospital (range = 6–146).

#### Harm

Almost half of the complaints in this sample reported no harm (*n *= 308, 48%). However, 12 (2%) of the complaints recorded catastrophic harm, i.e. permanent injury or death. A summary of the harm within complaints can be found in Table 1.

**Table 1:** Descriptive statistics from present analysis and comparison to UK data

HCAT domains and categories	Present study, Ireland	Gillespie and Reader, UK
	*N* (%)	*N* (%)
Total complaints	641	1110
Total complaints issues	1308	2047
Clinical		
*Quality*	189 (14%)	328 (16%)
*Safety*	160 (12%)	340 (16%)
Management		
*Environment*	115 (9%)	192 (9%)
*Institutional processes*	390 (30%)	523 (25%)
Relationships		
*Listening*	92 (7%)	133 (6%)
*Communication*	180 (14%)	259 (12%)
*Respect and patient rights*	182 (14%)	274 (13%)
Severity		
Low	292 (22%)	558 (27%)
Medium	726 (56%)	1030 (50%)
High	287 (22%)	486 (23%)
Uncoded	3 (0%)	NA
Stage of care		
1. Admissions	322 (25%)	353 (17%)
2. Examination/diagnosis	233 (18%)	443 (21%)
3. Care on the ward	370 (28%)	465 (22%)
4. Operation and procedures	78 (6%)	227 (11%)
5. Discharge	68 (5%)	310 (15%)
6. Other	171 (13%)	135 (7%)
Multiple stages	62 (5%)	75 (4%)
Not clear/uncoded	4 (0%)	66 (3%)
Harm	(Per complaint)	(Per complaint)
0—No harm reported	308 (48%)	409 (37%)
1—Minimal	112 (17%)	48 (4%)
2—Minor	114 (18%)	248 (22%)
3—Moderate	58 (9%)	152 (14%)
4—Major	28 (4%)	163 (15%)
5—Catastrophic	12 (2%)	90 (8%)
Unclear/not coded	9 (1%)	0 (0%)
Hot spots	Examination/diagnosis	Examination/diagnosis
	Care on ward	Care on ward
	Operation/procedure	Operation/procedure
Blind spots	Entry/exit	Entry/exit
	Systemic problems	Systemic problems
	Errors of omission	Errors of omission

#### Categories

All HCAT categories were represented in the complaints. Institutional processes problems made up 30% of all issues (*n *= 390), with quality (*n *= 189, 14%), communication (*n *= 180, 14%) and respect and patient rights (*n *= 182, 14%) representing the next most frequently mentioned categories. Table 1 details the distribution across the categories.

#### Severity

The majority of issues were classified as medium severity (*n *= 726, 56%). Table 1 presents a breakdown of all severity ratings.

#### Stage of care

The highest proportion of complaints issues (*n *= 370, 28%) referred to problems while the patients were receiving care on the ward (Stage 3 of the HCAT). This was closely followed by Stage 1, Admissions (*n *= 322, 25%). Table 1 presents a breakdown of the stages of care.

### Inter-rater reliability

Gwet’s AC1 across all aspects of the HCAT was 0.93 (CI = 0.92–0.94, *P* < 0.05). This was consistent with the findings of the original study by Reader and Gillespie in the UK using the HCAT, which had reliability ranging from 0.69 to 0.91 across categories [5].

### Hot spots

**Figure 1:**
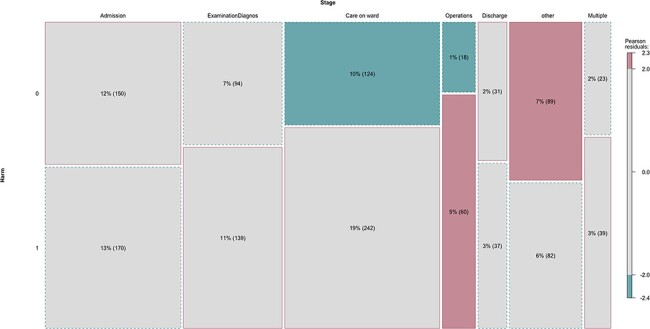
Hot spots for harm by stages of care.

The relationship between harm and stage of care as calculated using the chi-squared test was significant (*X^2^* (6, *n *= 1308) = 32.27, *P* < 0.05). This indicated that there were hot spots for harm, which were identified at three stages—care on the ward, examination and diagnosis and operations and procedures. There was also a hot spot when issues contained multiple stages of care. Figure 1 represents visually the hot spots at each of the stages of care. Blue boxes/a blue border indicates that the observed number of complaints issues at that point were fewer than expected, with red boxes/red border indicating that the observed number was more than expected. The colour of a box indicates the magnitude of the difference between the expected numbers of complaint issues per stage and the actual observed number of issues per stage. For example, a red box has more than expected complaints issues at that stage of care, and the value is farther from the expected value than a grey box with a red border. A blue box has fewer than expected complaints issues at that stage, and this number is further from the expected value than a grey box with a dotted blue border.

### Blind spots

Blind spots were identified at admissions and discharge and implicitly for errors of omission. Finally, a blind spot for systemic problems across the patient care pathway was identified using logistic regression. The model indicated that as the number of issues and stages per complaint increased, so too did the likelihood that harm would be reported within a complaint. Table 2 presents the model.

**Table 2:** Full logistic regression model for blind spots for harm

Coefficients	Estimate	Std error	*Z*-value	*P*
Intercept	−1.12	0.21	−5.34	<0.005*
Number of issues	0.36	0.09	3.92	<0.005*
Number of stages	0.35	0.18	1.96	0.0502

* Significant at a *P* < 0.005 level.

### Comparison with UK complaints analysis

Table 1 presents a comparison with the UK study of complaints in hospitals. The distribution of problems across categories, stages of care and severity levels were broadly similar across the two countries. The same hot spots and blind spots also emerged from the RoI and UK analyses.

## Discussion

### Statement of principal findings

Our analysis of complaints about hospital care using the HCAT allowed us to identify areas where harm was frequently reported by patients or family members (hot spots), and points in care where patients reported issues that staff could not see (blind spots). Hot spots occurred when patients were receiving care on the ward, during the examination and while undergoing a procedure. Blind spots included the boundaries of care and when patients experienced problems across the entire care pathway. Such data provide clear direction for where interventions are required to improve patient safety and quality of care. It also highlighted that the findings of the HCAT analysis of complaints about hospital care in the RoI are broadly comparable to those in the UK.

### Strengths and limitations

This study has several strengths and limitations. Strengths include that the study adds to the growing literature on the benefits of the HCAT for identifying issues within patient complaints [7, 12]. Second, this study reported high reliability for the HCAT in an Irish context, supporting existing data on the reliability of this tool [5, 9]. Third, this study involved multiple researchers and coders applying the HCAT, which reflects best practices [18]. Finally, the sample size achieved by this study was sufficient to provide a margin of error of only 2%, with a 95% confidence level [19].

Despite these strengths, there were also a number of limitations. Firstly, the sample size, while sufficient, restricted the complexity of analyses that were possible with some variables. Sampling complaints across a longer time frame could avoid this issue in future. The onset of the COVID-19 pandemic also inhibited the collection of complaints for this study, with many health service staff being redeployed to tackle the emerging public health crisis [20]. Further, a limitation of complaints research in general is that they represent only a small subsample of patients, who are willing and able to complain. Others may not be in a position to complain due to a lack of agency or knowledge or indeed out of fear of negative consequences to their care [21]. Finally, this study considered pre-COVID complaints only, and only complaints within an Irish and UK setting. Further research should explore how complaints may have changed since the beginning of the pandemic, and how the specific context of other countries may impact the categories that emerge from complaints.

### Interpretation within the context of wider literature

The hot spots and blind spots that emerged from the complaints about care in the RoI were similar to those that were reported in the UK [12]. To date, no other study has reported on hot spots and blind spots; however, these metrics could be vital for future quality improvement work. Such analyses could support a focus on areas in care that may require quality improvement. Complaints are an existing source of data in many healthcare settings and could be used to improve care where it is needed the most urgently [22]. Identifying areas such as these hot spots where harm is likely to occur is important for quality improvement and would allow for a prospective approach to improvement, rather than the retrospective approach taken by many other metrics [23]. Examples of this could include sharing the findings of a complaints analysis with stakeholders to develop and apply interventions. The UK and RoI health systems are relatively similar in structure [24, 25], which may explain why there were similar hot spots and blind spots within the complaints in both jurisdictions. It would be interesting to conduct similar analyses in other healthcare systems to determine whether the same, or different, hot spots and blind spots emerge.

While this study analysed complaints about hospitals in the RoI, the proportions of complaints issues that were assigned to each HCAT category were broadly similar to the UK [12]. In both studies, the ‘institutional processes’ category emerged most frequently. This seems to hold across other countries that have applied the HCAT [6–8] and highlights that complaints from individual patients can be indicative of system-level issues [22]. Understanding how patient complaints can shed light on management issues within a hospital system will be vital for providing evidence for interventions at this structural level [3, 12].

Harm was reported in over half of the complaints analysed from hospitals in the RoI. This is a key finding and links directly to the widely researched issue of iatrogenic harm in healthcare [26]. Identifying harm to patients using complaints could support staff-based measures of harm and add to our understanding of how this might be manifested [3, 23, 27]. The levels of harm reported in the RoI complaints were broadly similar to the UK [12]. It is also interesting that a large number of complaints reported no harm, which could be indicative of near-miss incidents that are perceived by patients and may not be captured in other staff-based metrics such as incident reports [3]. Future work should explore how complaints could be linked with other metrics to improve quality and safety in hospital care [28].

### Implications for policy, practice and research

This study has implications for policy, practice and research into patient complaints. It highlights the reliability of the HCAT, supporting the use of the tool to analyse patient complaints. The HCAT, and the taxonomy from which it was developed, is increasingly being used internationally to classify patient complaints in different settings (e.g. tertiary hospitals, maternity care and general practice [6, 8, 13, 29]). It has also been translated into Danish, and reliability has been found to hold in the translated version [10], as well as when used on compensation claims in Denmark [11]. It also exemplifies the benefits of analysing existing complaints data in a structured manner, in that any recommendations for improvements are evidence-based and reliable [22, 30]. However, complaints are only one data source. It is recommended that future research should consider how complaints can be linked with other methods of measuring and monitoring quality and safety in order to obtain a broader understanding of issues in healthcare than can be achieved by considering these data in isolation [28]. It could also be useful to examine compensation claims in the Irish healthcare system in a future study in a similar manner to the work emerging from Denmark [11].

Related to the previous point, there is arguably little point in analysing complaints data if it is not then used to bring about improvement. These data should be used not only to identify areas for improvement but also to support the design and evaluation of interventions to improve patient safety and quality of care [31]. It is suggested that the analysis of the hot spots and blind spots may be of particular use in identifying the focus of potential interventions and that a co-design approach with stakeholders could be used to achieve this, as with other areas of patient safety [32]. In a global healthcare system with entrenched resourcing and funding issues [33], using a tool such as the HCAT to prioritize interventions for patient safety and quality improvement has the potential to be particularly useful for implementing organizational change.

## Conclusions

The HCAT is a reliable tool when applied to the analysis of complaints about hospital care in the RoI. This study highlights the promise of the HCAT as a tool for the analysis of complaints across different healthcare systems, with similar results emerging in the RoI and the UK. The value of the HCAT for identifying hot spots and blind spots as areas for improvement is also supported by this study. However, for the analysis of complaints to be more than an academic exercise, it is important that moving forward, findings are used to drive the implementation of improvement interventions to address the issues identified.

## Data sharing statement

Data will be made available by the authors on reasonable request.
